# ﻿Review and redescription of species in the *brasiliana* group of Smicridea (Rhyacophylax) (Trichoptera, Hydropsychidae, Smicrideinae): exploration of the utility of geometric morphometrics as a method for delimitation and characterization of species in the genus

**DOI:** 10.3897/zookeys.1111.80961

**Published:** 2022-07-11

**Authors:** Julieta V. Sganga, Daniela E. Sganga, Mónica S. Iglesias

**Affiliations:** 1 Departamento de Biodiversidad y Biología Experimental, Facultad de Ciencias Exactas y Naturales, Universidad de Buenos Aires, Ciudad Universitaria, Pabellón II, C1428 EHA, Buenos Aires, Argentina Universidad de Buenos Aires Buenos Aires Argentina; 2 National Institute of Aquatic Resources, Technical University of Denmark, Kgs. Lyngby, Denmark Technical University of Denmark Lyngby Denmark

**Keywords:** Forewing shape, male genitalia, Neotropical, Smicridea (Rhyacophylax) atrobasis, Smicridea (Rhyacophylax) nanda, Smicridea (Rhyacophylax) vermiculata, Smicridea (Rhyacophylax) weidneri

## Abstract

The *Smicrideabrasiliana* species group includes five species distributed in northeastern Argentina and Brazil: Smicridea (Rhyacophylax) brasiliana (Ulmer), S. (R.) weidneri Flint, S. (R.) vermiculata Flint, S. (R.) arobasis Flint, and S. (R.) nanda Flint. The original descriptions of these species and their placement in the *brasiliana* species group were mainly based on the morphology of the male genitalia. However, the fine structure of the internal sclerites of the phallus, which proved to be useful for species delimitation, was not analyzed at the time. In this contribution, we provide a detailed description of the male genitalia and the morphology of the head, and analyze the shape of the wings using geometric morphometrics. The analyzed species can be easily differentiated by the shape of the phallus, especially by the structure of the internal sclerites, the shape of the head in dorsal view, and the shape of the cephalic setose warts. Furthermore, the geometric morphometric approach allowed their separation through the wing shape. The preliminary analysis of these features suggests that the *brasiliana* species group is not natural but its monophyly should be further tested within the framework of a phylogenetic analysis of all the species of the subgenus Rhyacophylax.

## ﻿Introduction

*Smicridea* is the only genus of Smicrideinae present in the Neotropical region (Schefter 1996; Flint et al. 1999). It is very diverse, represented by 255 described species grouped in two subgenera, Smicridea (Smicridea) McLachlan with 145 species and Smicridea (Rhyacophylax) Müller with 110 species (Holzenthal and Calor 2017; Alves et al. 2018; Mey and Ospina-Torres 2018; Sganga and Gibon 2018; Gibon and Sganga 2019; Rázuri-Gonzales and Armitage 2019; Vilarino et al. 2019; Desiderio et al. 2021; Queiroz et al. 2021; Santana et al. 2021). The taxonomy of *Smicridea* adult males has been studied extensively over the years, through the description of single species or the fauna of large geographic areas (e.g., Flint 1974). In the last decade approximately 74 new *Smicridea* species have been described (Albino et al. 2011; Rueda Martín and Sganga 2011; Oláh and Johanson 2012; Alves et al. 2018; Mey and Ospina-Torres 2018; Sganga and Gibon 2018; Gibon and Sganga 2019; Rázuri-Gonzales and Armitage 2019; Vilarino et al. 2019; Desiderio et al. 2021; Queiroz et al. 2021; Santana et al. 2021) but neither subgenus has been reviewed.

The Smicridea (Rhyacophylax) brasiliana (Ulmer, 1905) species group currently contains five species: *S.brasiliana*, *S.weidneri* Flint, 1972, *S.vermiculata* Flint, 1978, *S.atrobasis* Flint, 1983, and *S.nanda* Flint, 1983, that are distributed in northeastern Argentina and Brazil (Holzenthal and Calor 2017). This species group was established by Flint (1983) who did not provide a set of defining characters for the group but discussed the features that allowed the differentiation of these species (mainly the coloration, the presence of processes at the tip of the phallus, the shape of the internal sclerites, and the morphology of the tenth tergum and the inferior appendages). The relationships between these species were established by Flint in their original descriptions (Flint 1972, 1978, 1983). The identification of *Smicridea* species has long been based on the male genitalia, especially the structure of the tenth tergum and the phallus, allowing the delimitation of several species groups in both subgenera. These groups were never formally defined (following the principle of monophyly) but created to include species with similar characteristics. Because of this missing framework, several species in both subgenera were never placed in a species group. Oláh and Johanson (2012) summarized some of these groups, listed their defining characters, and the species included in them, in order to provide a framework to include their new species. However, a comprehensive work including a review and phylogenetic analysis of all *Smicridea* species, and a test of the validity of these species groups is lacking.

The morphology of the male genitalia has been extensively used for the delimitation of species in the order Trichoptera as a whole. Other characteristics of the adult morphology, such as the shape of the antennae and palps, the presence of ocelli, spur formula, shape and distribution of setose warts, and wing venation are usually used to identify families and genera (Holzenthal et al. 2007). In the genus *Smicridea* the structure of the internal sclerites of the phallus has been proven to be useful for the differentiation of species in the subgenus Rhyacophylax, mainly in closely related ones (e.g., Rocha et al. 2016; Santana et al. 2021). Despite its importance, this character has not been analyzed in detail in the early descriptions of *Smicridea* species, which is the case for *S.brasiliana*, *S.nanda*, and *S.weidneri*. A few authors have analyzed non-genital characters for the identification of *Smicridea* species. Oláh and Johanson (2012) and Sganga and Gibon (2018) used the maxillary palp formula to represent the length ratio of the 5 palp articles. More recently, Vilarino et al. (2019) explored the use of new characters to evaluate species delimitation such as the presence and shape of head setose warts and sutures, eye size, and forewing forks. All these features were variable among the species described and represent a source of characters for future phylogenetic analysis.

In recent decades, the number of geometric morphometric studies in insects has increased in the literature. This methodology became a powerful tool to detect minimal shape variations which often are undetectable by traditional morphological studies and emphasizes differences between groups (Villemant et al. 2007). It is usually applied to distinguish species (Baylac et al. 2003; Lorenz et al. 2017; Simões et al. 2020), identify population structure (Kiyoshi and Hikida 2012; Kamimura et al. 2020), sexual dimorphism (Gushki et al. 2018), study morphological evolution during ontogeny (Springolo et al. 2021), and map phylogenetic hypotheses (Huang et al. 2020), among others. Studies based on the taxonomic delimitation of species that are difficult to solve by traditional anatomical methods have been carried out in various insect taxa (Sábio et al. 2014), many of them based on wing geometry (Kiyoshi and Hikida 2012; Shimabukuro et al. 2016; Huang et al 2020; Simões et al. 2020). The wings of the species of Smicridea (Rhyacophylax) are very conserved in the arrangement of their veins, which makes them an excellent material to investigate interspecific variations for the delimitation of species.

In the present work, we aimed to redescribe the species in the *Smicrideabrasiliana* group offering a detailed description of the genital segments, especially the phallus, and provide non-genital characters for their identification. Additionally, we tested the use of geometric morphometrics for species delimitation. Integrative taxonomic investigations, which include traditional tools together with modern methodologies, are increasingly being implemented to solve species delimitation problems (González et al. 2019). Geometric morphometrics techniques show high performance in this task (Mutanen and Pretorius 2007). This work represents the first study that incorporates the geometric morphometric approach to the taxonomy of the order Trichoptera, in particular the genus *Smicridea*.

## ﻿Materials and methods

Specimens of *Smicrideabrasiliana*, *S.nanda* and *S.weidneri* housed in the National Museum of Natural History, Smithsonian Institution, Washington DC (**USNM**) were examined. Those specimens were identified and loaned by Dr. Oliver Flint Jr. Additionally, we borrowed specimens of *S.atrobasis* and S. (R.) vekona from the Facultad de Humanidades y Ciencias, Universidad de la República (Uruguay, **FHCM**) and the Instituto de Biodiversidad Neotropical, CONICET-Universidad Nacional de Tucumán, (Argentina), respectively. Specimens of the other species treated herein were collected in Salto Encantado Provincial Park and Forest Refuge and research center Antonia Ramos (Misiones province, Argentina).

The samples were collected in December 2004 and November 2013 using light and Malaise traps. The specimens obtained were fixed and preserved in 80% EtOH. Voucher specimens were deposited at the Museo Argentino de Ciencias Naturales (Buenos Aires, Argentina).

For identification and illustration of the specimens the abdomen was cleared using a hot 10% NaOH solution. Then the cuticle was rinsed in distilled water, neutralized with acetic acid, and mounted in a dish with glycerin for observation. Line drawings of the genital structures were produced using a camera lucida attached to a microscope. Line illustrations of the heads were constructed using photographs as templates, which were obtained with a digital camera fixed to a stereomicroscope. All the images were digitalized with Adobe Illustrator (v. 15.0.0 Adobe Systems Inc.).

**Figure 1.**
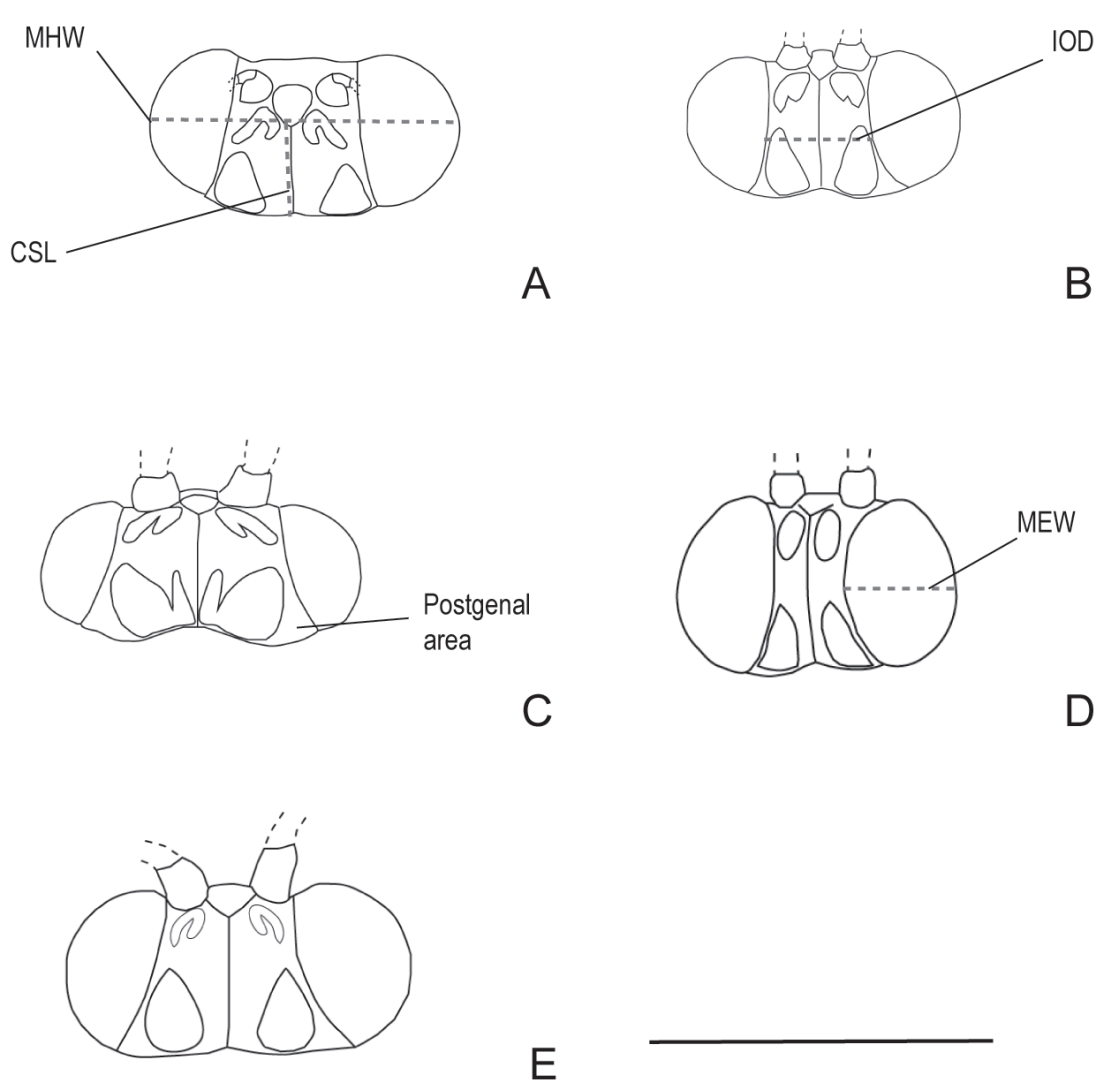
Heads in dorsal view of the species of the *brasiliana* group **A**Smicridea (Rhyacophylax) brasiliana**B**S. (R.) weidneri**C**S. (R.) vermiculata**D**S. (R.) atrobasis**E**S. (R.) nanda. Abbreviations: CSL length of the coronal suture, IOD interocular distance, MEW maximum eye width, MHW maximum head width. Scale bar: 1 mm.

For the description of the heads the following distances were measured (Fig. 1):

**IOD** interocular distance;

**CSL** length of the coronal suture;

**MEW** maximum eye width;

**MHW** maximum head width.

All the measures were taken using a stereomicroscope with a graduated eyepiece. The terminology used by Albino et al. (2011) was followed for the description of the male genitalia, the one from Wells and Neboiss (2018) for the setose warts, and the one from Oláh and Johanson (2007) for the cranial areas.

For the morphometric analysis, all the species included in the *brasiliana* group were used along with five additional species from the same subgenus, in order to increase the discriminatory power of the methodology.

**Figure 2.**
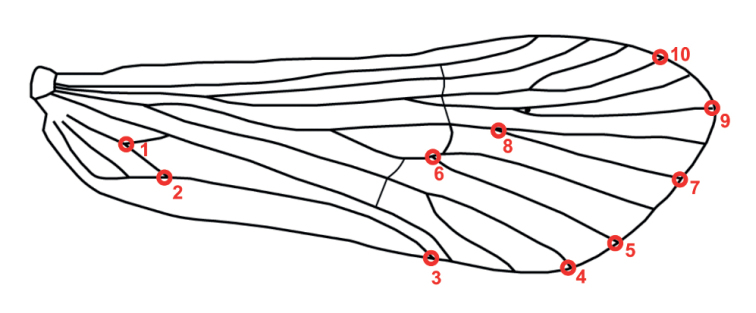
Forewing of Smicridea (Rhyacophylax) mesembrina showing the location of the selected landmarks (1–10).

The left forewings of males (*n* = 154) of Smicridea (Rhyacophylax) mesembrina (Navás, 1918) (*n* = 21), *S.weidneri* (*n* = 16), *S.vermiculata* (*n* = 22), S. (R.) spinulosa Flint, 1972 (*n* = 18), *S.atrobasis* (*n* = 18), S. (R.) vekona Oláh & Johanson, 2012 (*n* = 19), S. (R.) pampeana Flint, 1980 (*n* = 18), S. (R.) unguiculata Flint, 1983 (*n* = 20), *S.nanda* (*n* = 1), and *S.brasiliana* (*n* = 1) were dissected. Then, the removed wings were extended and mounted on a slide, using alcohol as medium, and covered with a coverslip. The alcohol was left to evaporate before taking photographs with a digital camera fixed to a stereomicroscope (two photographs were taken of each wing). Cartesian coordinates of ten landmarks of each wing (Fig. 2) were digitized using tps-UTILS v. 1.38 (Rohlf 2006a) and tps-DIG v. 2.05 (Rohlf 2006b). The landmark configurations were scaled, translated and rotated using the GLS Procrustes superimposition method (Bookstein 1991) using the MorphoJ software v. 1.06d (Klingenberg 2011) and subsequently a thin-plate spline analysis was performed allowing the visualization of shape differences as deformation.

Permutation tests for distances by species pairs (20,000 rounds of permutations) for the Mahalanobis (Table 1) and Procrustes distances were performed. Then the percentage of correct reclassification by pairs of species was calculated from the cross-validation procedure.

Canonical variate analysis (**CVA**) was performed on aligned landmark coordinates and the specimens were reclassified to each species (jackknife method) to evaluate the effectivity of the discriminant analysis for assigning them to their own group using the software Past v. 4.02 (Hammer et al. 2001).

The species *S.nanda* and *S.brasiliana* were excluded from all the statistical tests mentioned above due to an insufficient number of specimens.

A principal component analysis (**PCA**) with the consensus configurations of the species of the *brasiliana* group (*S.brasiliana*, *S.weidneri*, *S.vermiculata*, *S.atrobasis*, and *S.nanda*) was performed. In addition, the thin plate spline method was used to illustrate the transformations of the wing shapes compared to the consensus wing shape of the group. Mahalanobis distances between the mean shapes of each species of the *brasiliana* group were used to construct a dendrogram using the Unweighted pair-group method with arithmetic mean (**UPGMA**) with the software Past v. 4.02 (Hammer et al. 2001).

For wing size analysis, centroid size (**CS**) was used as a measure of size and was computed as the square root of the sum of squared distances from all landmarks to the centroid of the landmarks configuration (Bookstein 1991). The CS variation for each species is shown with a violin-plot. Differences in CS among species were assessed through a Kruskal-Wallis test and a posteriori pairwise test. *Smicrideananda* and *S.brasiliana* were not included in this analysis due to insufficient number of specimens, but the CS of both species are shown in the plot.

## ﻿Systematics

### ﻿Family Hydropsychidae Curtis, 1835

**Subfamily Smicrideinae Schefter, 1996**

**Genus *Smicridea* Mclachlan, 1871**

#### Subgenus Rhyacophylax Müller, 1879

##### ﻿ Smicridea (Rhyacophylax) brasiliana

Taxon classificationAnimaliaTrichopteraHydropsychidae

(Ulmer, 1905)

E231C087-F8D8-5738-BFC1-8C779F9B91C1

Figs 1A , 3A–E

Smicridea (Rhyacophylax) brasiliana (Ulmer), 1905: 107 [as Rhyacophylaxbrasilianus]. Weidner 1964: 97 [lectotype]. Flint 1966: 7 [invalid lectotype, misidentification]; 1972: 238 [discussion of lectotype]. Paprocki et al. 2004: 9 [checklist]; Paprocki and França 2014: 32 [checklist]. Holzenthal and Calor 2017: 165 [catalog].

###### Material examined.

Argentina • 1 male; Misiones, Río Iguazú, camp. Nandu; 25 Feb. 1973; OS Flint Jr. det.; USNM.

Flint (1972) examined the type series of this species from the Ulmer collection (housed at the Zoologisches Museum Hamburg) where he found two mixed species, Smicridea (Rhyacophylax) brasiliana and another closely related species that he described as S. (R.) weidneri. The specimen we used for this redescription was collected in 1973 in Misiones province (Argentina) and identified by Dr. Flint. This specimen was borrowed from the USNM.

###### Description.

**Adult male.** General color of the body light brown. Length of the forewings: 6.3 mm (*n* = 1). Coloration of the forewings similar to the body, with a subapical transverse, sinuous, white stripe, and a white, rectangular spot at midlength of the costal margin.

***Head*** (Fig. 1A). In dorsal view rectangular, transverse. Mesal margins of the eyes, in dorsal view, parallel, postgenal areas reduced. Interocular area rectangular, wider than long. Interocular distance 2.2 × shorter than MHW. Coronal suture 2/3 × shorter than IOD. Eyes lightly produced anteriorly, maximum eye width 3.3 × shorter than MHW. Anterolateral setose warts present, subtriangular, bifid posteriorly, mesal lobe shorter than the lateral. Posterior setose warts subtriangular. Maxillary palps missing.

**Figure 3.**
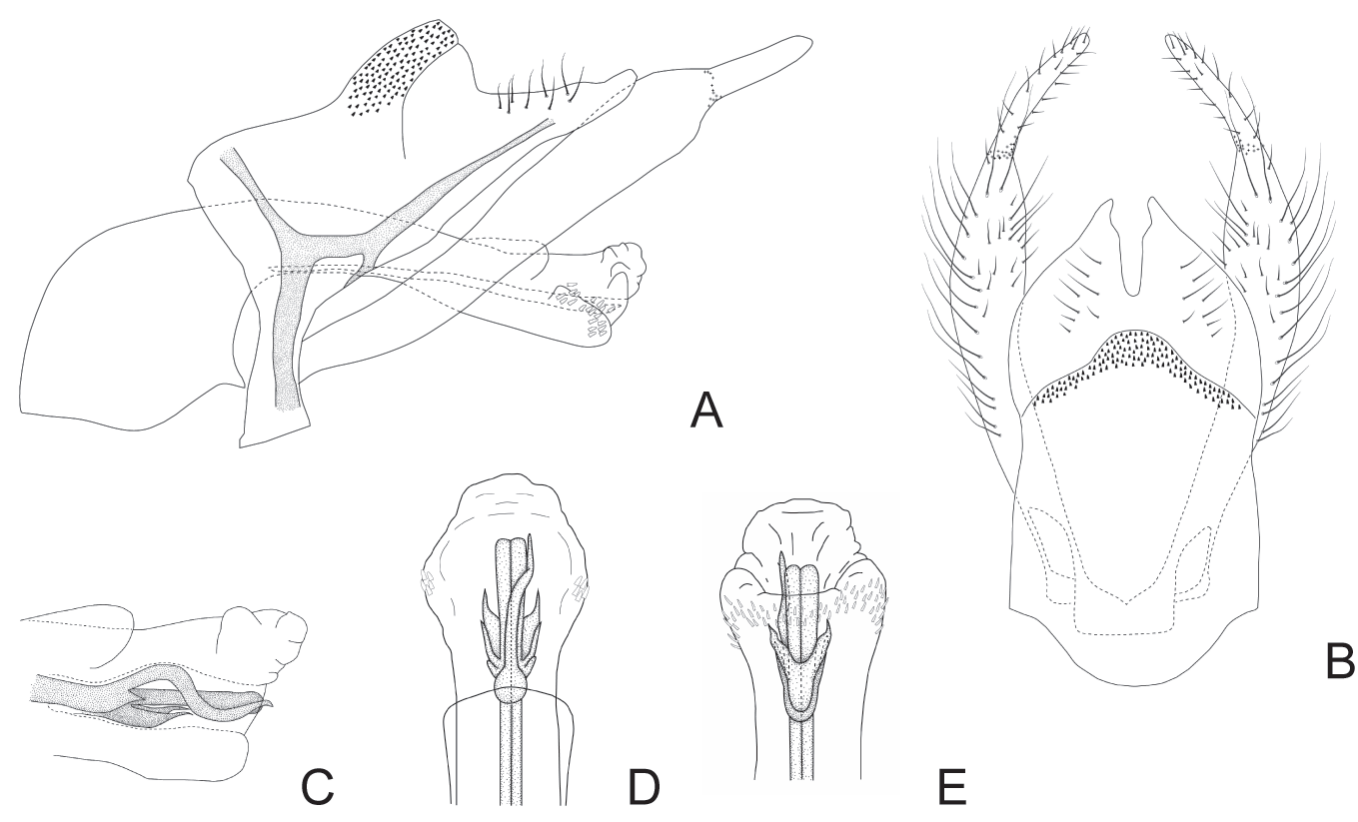
Male genitalia of Smicridea (Rhyacophylax) brasiliana**A** segments IX, X, inferior appendages and phallus, lateral view **B** segments IX, X and inferior appendages, dorsal view **C** tip of the phallus, lateral view (lateral spines removed) **D** tip of the phallus, dorsal view **E** tip of the phallus, ventral view.

***Male genitalia*.** Anterolateral margin of segment IX rounded and produced (Fig. 3A). Tergum of segment X triangular in lateral view, apex rounded, dorsal and ventral margins straight, the ventral one with a sclerotized H-shaped area directed anteriorly through segment IX (Fig. 3A); in dorsal view divided mesally into two subtriangular hemitergites with apex subacute; internal margin of each hemitergite straight, with a concavity subapically (Fig. 3A, B). Inferior appendages with two articules, setose, curved mesally in dorsal view; basal article narrow for a short distance proximally, from where it widens to the apex; apical article narrow, short, with rounded apex (Fig. 3B). Phallus with long and tubular phallobase; basal portion broad, forming an angle of ~ 90° with distal part, which is slightly curved and with widened apex (Fig. 3A, C); dorsal periphallic cap present subapically; apex of the phallus with a row of small spines extending from one side to the other ventrally, in dorsal view slightly produced laterally (Fig. 3A, C–E). Internal sclerotized section of ejaculatory duct long and straight in lateral view (~ 2/3 the phallobase length), in dorsal view longitudinally divided in two (Fig. 3E); distal end with an elongate, pointed dorsal plate, that bends upwards, then ventrad to the left at mid-length and upwards again, ending slightly beyond the tip of the ejaculatory duct; basally this plate bears a lateral spine; ventrally to the ejaculatory duct there are two spine-like plates and two lateroventral subrectangular plates that narrow posteriorly ending in a point (Fig. 3C–E). Endotheca simple.

###### Systematic considerations.

This species seems to be related to *S.weidneri* and *S.nanda*. Genitalically, these species share the presence two pairs of elongate sclerites, dorsal and ventrad to the ejaculatory duct, which take different forms in the three species. Additionally, the morphology of the setose warts of the head of these species is similar, with the anterolateral setose warts bifid and the posterior ones triangular. *Smicrideabrasiliana* can be distinguished by the presence of the elongate, sinuous, and pointed dorsal plate at the distal end of the ejaculatory duct, absent in the other two species, and the shape of the ventral plates that are spine-like, and the lateroventral ones that are subrectangular and pointed. Also, *S.brasiliana* has series of spines surrounding lateroventrally the end of the phallus, which are lacking in the other two species.

###### Distribution.

Argentina (new record), Brazil.

##### ﻿ Smicridea (Rhyacophylax) weidneri

Taxon classificationAnimaliaTrichopteraHydropsychidae

Flint, 1972

70CEEC3B-18D8-5E8D-A532-1CAC899C03A2

Figs 1B , 4A–D

Smicridea (Rhyacophylax) weidneri Flint, 1972: 238; 1966:8 [as brasilianus, distribution]. Marinoni and de Almeida 2000: 286 [distribution; biology]. Paprocki et al. 2004: 9 [checklist]. Sganga 2006: 142 [distribution]. Paprocki and França 2014: 37 [checklist]. Manzo et al. 2014: 166 [distribution]. Holzenthal and Calor 2017: 187 [catalog].

###### Material examined.

Argentina • 1 male; Misiones, Capiovy; 5 Apr. 1971; CM & OS Flint Jr. col.; paratype; USNM • 15 males; Misiones, Oberá, Centro de Investigación y Refugio de Selva Antonia Ramos, A° Ramos; 17 Nov. 2013; JV Sganga col.; light trap.

###### Description.

**Adult male.** Coloration of the body stramineous. Length of the forewings 4.5 mm (*n* = 16), coloration similar to that of the body, with two transverse, brown bands, one subapical, almost straight and the other sinuous, at midlength.

***Head*** (Fig. 1B). In dorsal view rectangular, transverse. Mesal margins of the eyes, in dorsal view, concave, postgenal areas small, triangular. Interocular area rectangular, longer than wide. Interocular distance 2.75 × shorter than MHW. Coronal suture 1.08 × longer than IOD. Maximum eye width 3 × shorter than MHW. Anterolateral setose warts present, oval, with a V-shaped notch posteriorly. Posterior setose warts subtriangular. Maxillary palp formula: I-II-IV-III-V.

**Figure 4.**
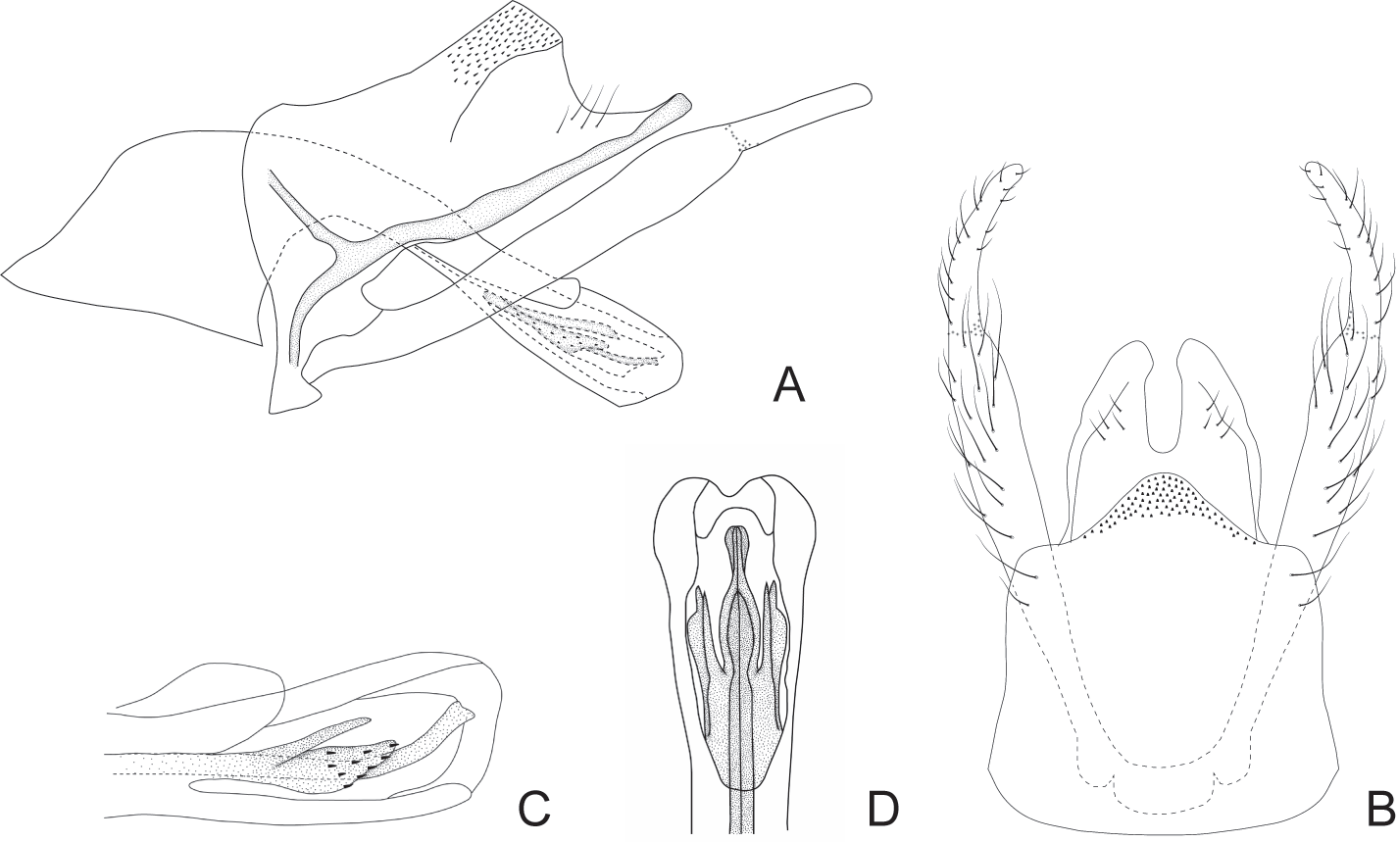
Male genitalia of Smicridea (Rhyacophylax) weidneri**A** segments IX, X, inferior appendages and phallus, lateral view **B** segments IX, X and inferior appendages, dorsal view **C** tip of the phallus, lateral view **D** tip of the phallus, dorsal view.

***Male genitalia*.** Anterolateral margin of segment IX slightly rounded. Tergum of segment X triangular in lateral view, dorsal and ventral margins straight, the ventral one with a sclerotized Y-shaped area directed anteriorly through segment IX (Fig. 4A); in dorsal view divided mesally into two triangular hemitergites, with apex rounded and mesal margins concave (Fig. 4B). Inferior appendages with two articles, basal article slightly widened distally, apical one curved mesad in dorsal view, slightly narrowing towards the apex, which is rounded (Fig. 4A, B). Phallus long, with a tubular phallobase; basal portion broad, forming an angle of ~ 90° with distal part, that is straight and with apex somewhat widened; dorsal periphallic cap present subapically (Fig. 4A). Internal sclerotized section of ejaculatory duct long and sinuous in lateral view (~ 2/3 the phallobase length), distal end curved upwards (Fig. 4A, C); in dorsal view longitudinally divided in two, apex spindle-shaped (Fig. 4D); in lateral view with two romboidal dorsolateral plates in the posterior half of the ejaculatory duct, with ventral and posterior margins serrated and with small spines on its distal surface; dorsal to these plates there are two wide spine-like plates directed posteriorly (Fig. 4A, C, D). Endotheca simple.

###### Systematic considerations.

This species seems to be closely related to *S.brasiliana* and *S.nanda* (see Systematic considerations section for *S.brasiliana*). *Smicrideaweidneri* can be identified by the spindle-shaped distal end of the ejaculatory duct, that is simple in the other two species, and the shape of the two pairs of plates, two rhomboidal, with ventral and posterior margins serrated, and covered with small spines, and two spine-like, wide, directed posteriorly.

###### Distribution.

Argentina, Brazil.

##### ﻿ Smicridea (Rhyacophylax) vermiculata

Taxon classificationAnimaliaTrichopteraHydropsychidae

Flint, 1978

ECFD3EFF-411B-5424-B34A-6E43F2019DF2

Figs 1C , 5A–D

Smicridea (Rhyacophylax) vermiculata Flint, 1978: 381. Marinoni and de Almeida 2000: 286 [distribution; biology]. Blahnik et al. 2004: 4 [distribution]. Paprocki et al. 2004: 9 [checklist]. Sganga 2006: 142 [distribution]. Calor 2011: 321 [checklist]. Paprocki and França 2014: 36 [checklist]. Holzenthal and Calor 2017: 186 [catalog].

###### Material examined.

Argentina • 27 males; Misiones, Oberá, Centro de Investigación y Refugio de Selva Antonia Ramos, A° Ramos; 17 Nov. 2013; JV Sganga col.; light trap.

###### Description.

**Adult male.** General coloration of the body brown. Length of forewings 4.5 mm (*n* = 22), coloration similar to that of the body, with a distinct transverse, white band subapically.

***Head*** (Fig. 1C). In dorsal view rectangular, transverse. Internal margins of the eyes, in dorsal view, convergent, postgenal areas triangular. Interocular area trapezoidal. Interocular distance 1.85 × shorter than MHW. Coronal suture 1.47 × shorter than IOD. Maximum eye width 4.35 × shorter than MHW. Anterolateral setose warts present, oval, bifid posteriorly, with mesal lobe shorter than the lateral. Posterior setose warts subtriangular, with a digitate mesal lobe. Maxillary palp formula: I-II-IV-III-V.

**Figure 5.**
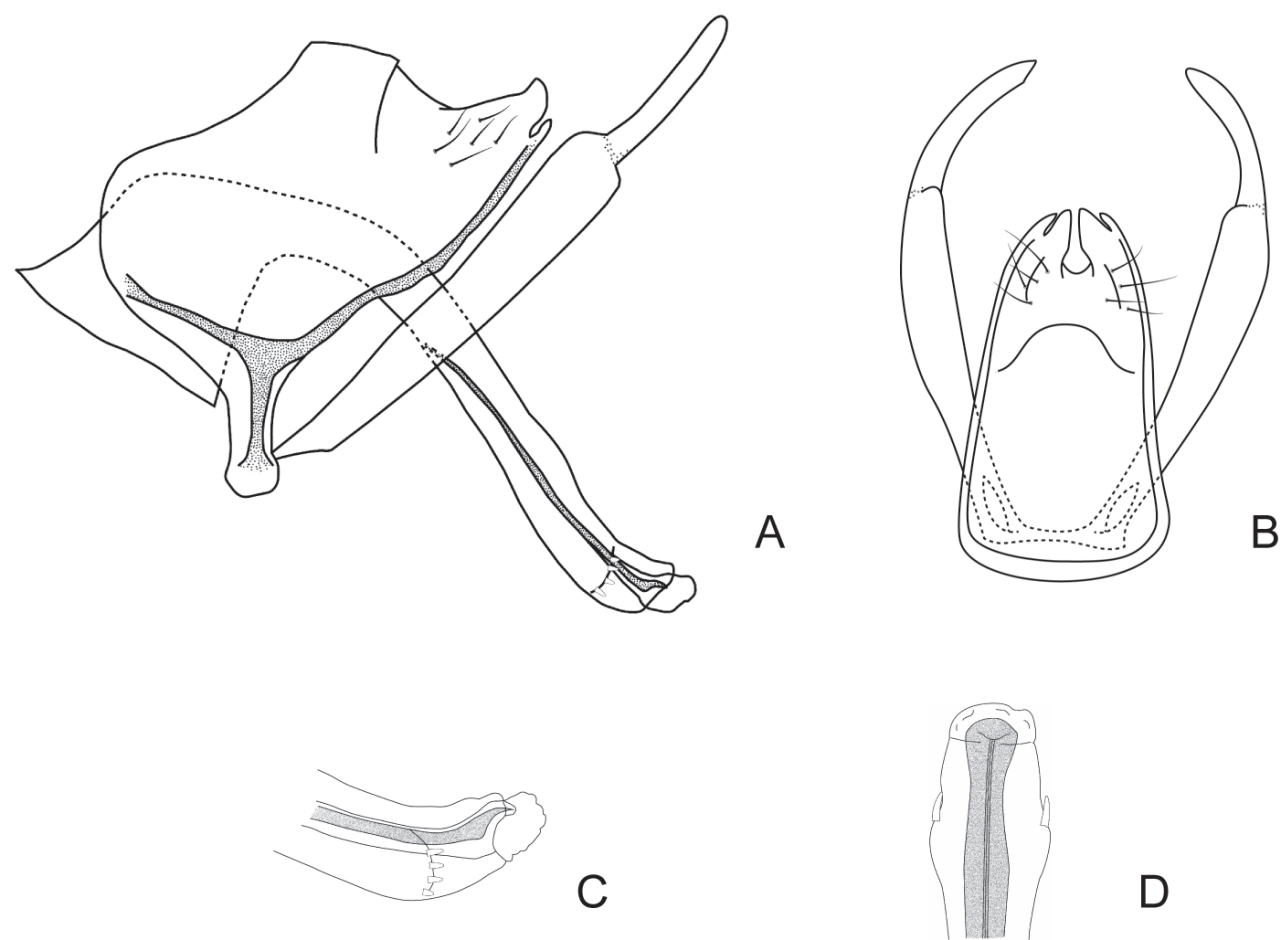
Male genitalia of Smicridea (Rhyacophylax) vermiculata**A** segments IX, X, inferior appendages and phallus, lateral view **B** segments IX, X and inferior appendages, dorsal view **C** tip of the phallus, lateral view **D** tip of the phallus, dorsal view.

***Male genitalia*.** Anterolateral margin of segment IX rounded, produced. Tergum of segment X triangular in lateral view, dorsal margin straight, ventral slightly rounded, with a subapical lobe, and a sclerotized Y-shaped area directed anteriorly through segment IX (Fig. 5A); in dorsal view divided mesally into two triangular hemitergites, with apex rounded and bearing a lateral lobe, mesal margins straight, with an anterior notch (Fig. 5B). Inferior appendages with two articules, basal article slightly widened distally, apical one curved mesad in dorsal view, apex pointed (Fig. 5A, B). Phallus long, with a tubular phallobase; basal portion broad, forming an angle of ~ 90° with distal part, which is very long and with apex slightly upturned (Fig. 4A); phallus bears subapically four spines mesoventrally on each side, which are directed posteriorly (Fig. 5A, C, D). Internal sclerotized section of ejaculatory duct long (~ 1/2 the phallobase length) and bent ventrad anteriorly in lateral view, distal end curved upwards and with a posterior concavity (Fig. 5A, C); in dorsal view longitudinally divided in two (Fig. 5D). Endotheca simple.

###### Systematic considerations.

This species seems to be related to S. (R.) dentifera Flint, 1983 and S. (R.) unguiculata. The three species have simple ejaculatory ducts and lateroventral spines at the apex of the phallus. The features that allow the differentiation of *S.vermiculata* are the position of the spines of the phallus (it has 4 mesoventral spines on each side directed posteriorly), the ejaculatory duct that is curved upwards and bears a posterior concavity, and the presence of the apicolateral lobe on tergum X.

###### Distribution.

Argentina, Brazil, Paraguay.

##### ﻿ Smicridea (Rhyacophylax) atrobasis

Taxon classificationAnimaliaTrichopteraHydropsychidae

Flint, 1983

A55D5855-437E-5B43-BAD3-CCF18D8EDFC7

Figs 1D , 6A–D

Smicridea (Rhyacophylax) atrobasis Flint, 1983: 63. Paprocki et al. 2004: 9 [checklist]. Sganga 2006: 142 [distribution]. Sganga and Angrisano 2005: 132 [distribution]. Rueda Martín and Sganga 2011: 2225 [♂; distribution]. Paprocki and França 2014: 32 [checklist]. Isa Miranda and Rueda Martín 2014: 200 [distribution]. Holzenthal and Calor 2017: 163 [catalog].

###### Material examined.

Uruguay • 22 males; Salto, Salto Grande; 19 Nov. 1955; a la luz, en la cascada; FHCM • 1 male; Artigas, río Uruguay, barra Arroyo Guaviyú; 22 Nov. 1954; CS Carbonell leg. (OS Flint Jr. det.) • 1 male; San Gregorio; 29 Nov. 1959; Carbonell, Mesa, San Martín leg. (OS Flint Jr. det.).

###### Description.

**Adult male.** Coloration of the body in alcohol stramineous. Length of forewings 5.4 mm (*n* = 18). We were not able to observe the coloration of the wings in the specimens preserved in alcohol due to discoloration of the cuticle through time, but Flint (1983) described it from dried specimens as follows: “forewings dark purplish black in basal quarter and in two transverse bands apicad, otherwise covered with golden hair”.

***Head*** (Fig. 1D). In dorsal view rectangular, transverse. Mesal margins of the eyes, in dorsal view, convex, postgenal areas reduced. Interocular area rectangular, narrow, longer than wide. Interocular distance 7.3 × shorter than MHW. Coronal suture 2.2 × longer than IOD. Eyes very prominent, maximum eye width 2.4 × shorter than MHW. Anterolateral setose warts present, elongate, oval. Posterior setose warts subtriangular. Maxillary palp formula: I, II-III, IV-V.

**Figure 6.**
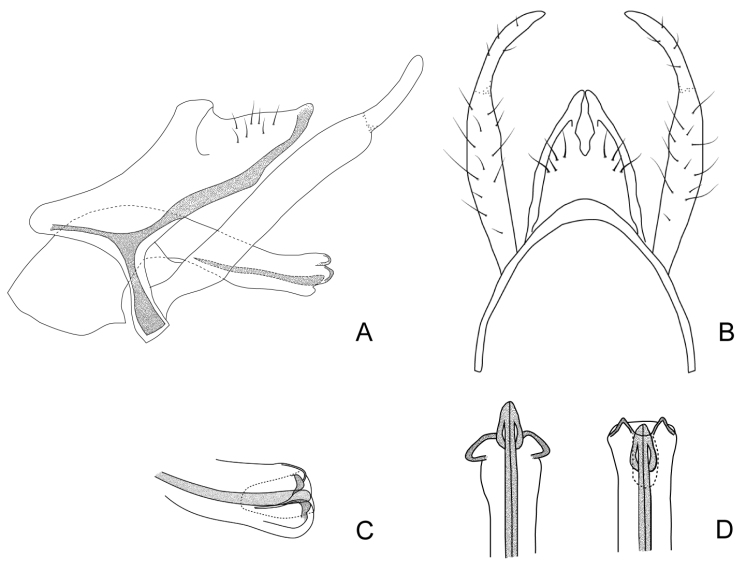
Male genitalia of Smicridea (Rhyacophylax) atrobasis**A** segments IX, X, inferior appendages and phallus, lateral view **B** segments IX, X and inferior appendages, dorsal view **C** tip of the phallus, lateral view **D** tip of the phallus, dorsal view (left evaginated, right invaginated).

***Male genitalia*.** Anterolateral margin of segment IX sinuous. Tergum of segment X triangular in lateral view, dorsal and ventral margins rounded, with a ventral sclerotized Y-shaped area directed anteriorly through segment IX (Fig. 6A); in dorsal view divided mesally into two triangular hemitergites, with apex pointed, mesal margins concave (Fig. 6B). Inferior appendages with two articules, basal article slightly widened distally, apical one curved mesad in dorsal view, apex pointed (Fig. 6A, B). Phallus long, with a tubular phallobase; basal portion broad, forming an angle of ~ 120° with distal part; apex broadened, ending in two laterodorsal and two lateroventral lobes, that become directed basad as the endotheca is everted (Fig. 6A, C, D). Internal sclerotized section of ejaculatory duct ~ 2/3 the phallobase length, straight, slightly upturned apically in lateral view (Fig. 6A, C); in dorsal view longitudinally divided in two, apex shaped like an arrowhead (Fig. 6D). Endotheca simple.

###### Systematic considerations.

This species seems to be closely related to *Smicrideamesembrina*. These two species bear apicolateral lobes on the phallus, but while the apex of the sclerotized section of the ejaculatory duct in *S.mesembrina* is simple, in *S.atrobasis* it is shaped like an arrowhead. Additionally, *S.atrobasis* has a very distinctive feature that is the prominent eyes and reduced interocular area.

###### Distribution.

Argentina, Bolivia, Brazil, Uruguay.

##### ﻿ Smicridea (Rhyacophylax) nanda

Taxon classificationAnimaliaTrichopteraHydropsychidae

Flint, 1983

F8AD2928-14D5-5E7D-98B8-AE461592E4E1

Figs 1E , 7A–D

Smicridea (Rhyacophylax) nanda Flint, 1983:65. Sganga 2006: 142 [distribution]. Holzenthal and Calor 2017: 177 [catalog].

###### Material examined.

Argentina • 1 male; Misiones, Río Iguazú, camp. Nandu; 25 Feb. 1973; OS Flint Jr. col.; paratype; USNM.

###### Description.

**Adult male.** General coloration of the body light brown. Length of forewings 6.8 mm (*n* = 1), coloration similar to that of the body, with a soft darkening on the crossveins and a pale, subapical, transverse band.

***Head*** (Fig. 1D). In dorsal view rectangular. Internal margins of the eyes, in dorsal view, concave, postgenal areas small, triangular. Interocular area trapezoidal. Interocular distance 2.6 × shorter than MHW. Coronal suture 1 × the length of IOD. Eyes slightly produced anteriorly, maximum eye width 3.25 × shorter than MHW. Anterolateral setose warts present, very subtle, oval, bifid posteriorly. Posterior setose warts subtriangular. Maxillary palp formula: I-II-III-IV-V.

**Figure 7.**
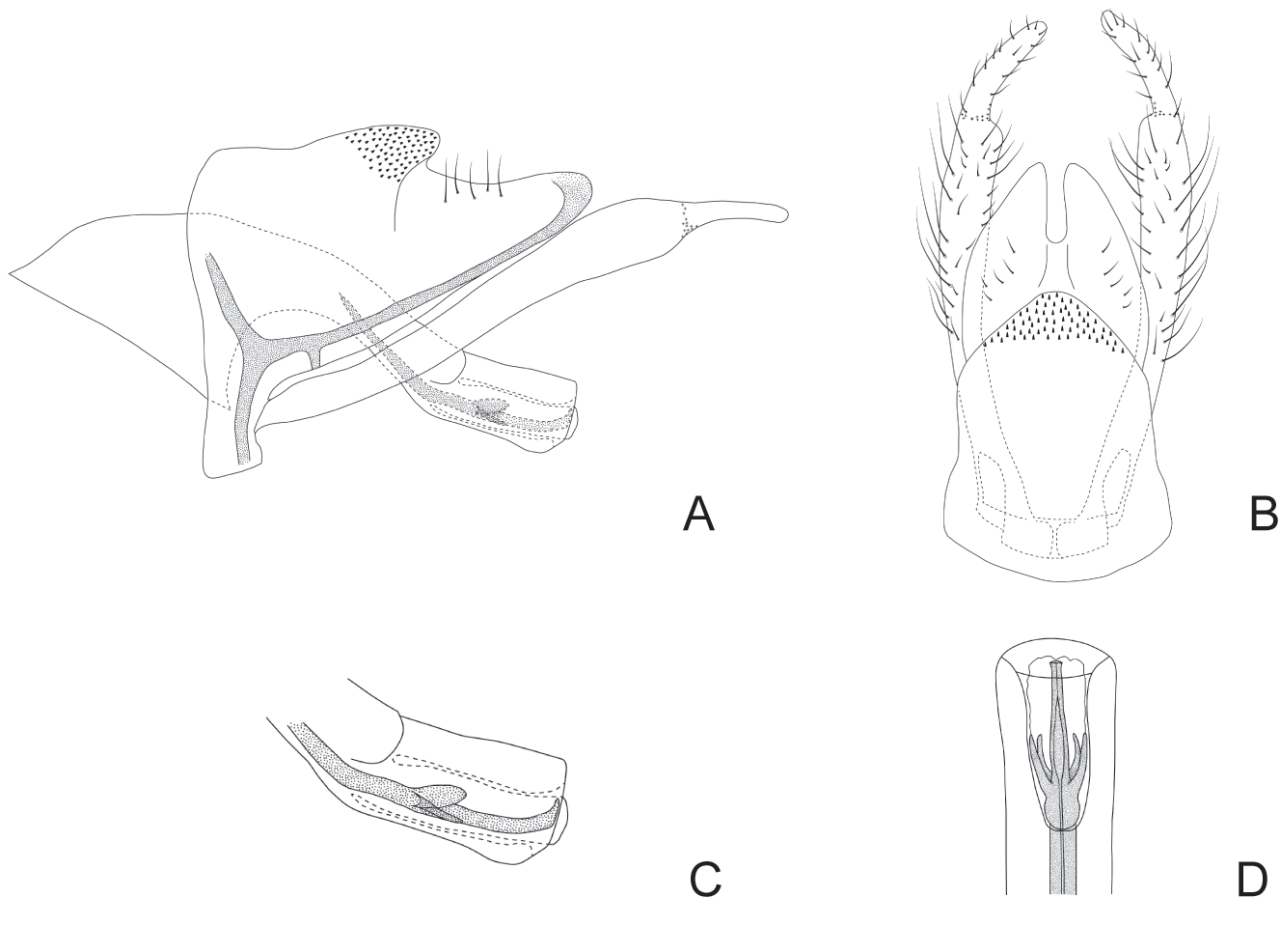
Male genitalia of Smicridea (Rhyacophylax) nanda**A** segments IX, X, inferior appendages and phallus, lateral view **B** segments IX, X and inferior appendages, dorsal view **C** tip of the phallus, lateral view **D** tip of the phallus, dorsal view.

***Male genitalia*.** Anterolateral margin of segment IX slightly rounded on the dorsal half (Fig. 7A). Tergum of segment X subtriangular in lateral view, with rounded apex, dorsal and ventral margins straight, ventral one with a sclerotized H-shaped area directed anteriorly through segment IX; in dorsal view divided mesally into two subtriangular hemitergites with rounded apex and mesal margins straight (Fig. 7B). Inferior appendages with two articules, curved mesally in dorsal view, basal article slightly widened distally, apical article narrow, short, apex pointed (Fig. 7A, B). Phallus with long and tubular phallobase; basal portion slightly broad, bending ventrad mesally, distal part straight (Fig. 7A); basal and dista parts of the phallus forming an angle of ~ 90°; dorsal periphallic cap present at midlength. Sclerotized part of ejaculatory duct curved dorsad at midlength; tip directed upwards in lateral view; with two dorsolateral elongated, oval plates in lateral view and two spine-like sclerites beneath them (Fig. 7A, C, D). Endotheca wrinkled (Fig. 7D).

###### Systematic considerations.

This species seems to be related to *S.brasiliana* and *S.weidneri* (see Systematic considerations section for *S.brasiliana*). *Smicrideananda* is characterized by the ejaculatory duct that is curved dorsad at midlength and distally upturned, and the shape of the two pairs of sclerotized plates: the dorsolateral ones elongated, oval in lateral view and the other two spine-like lying beneath them.

###### Distribution.

Argentina.

### ﻿Geometric morphometric analysis

**Figure 8.**
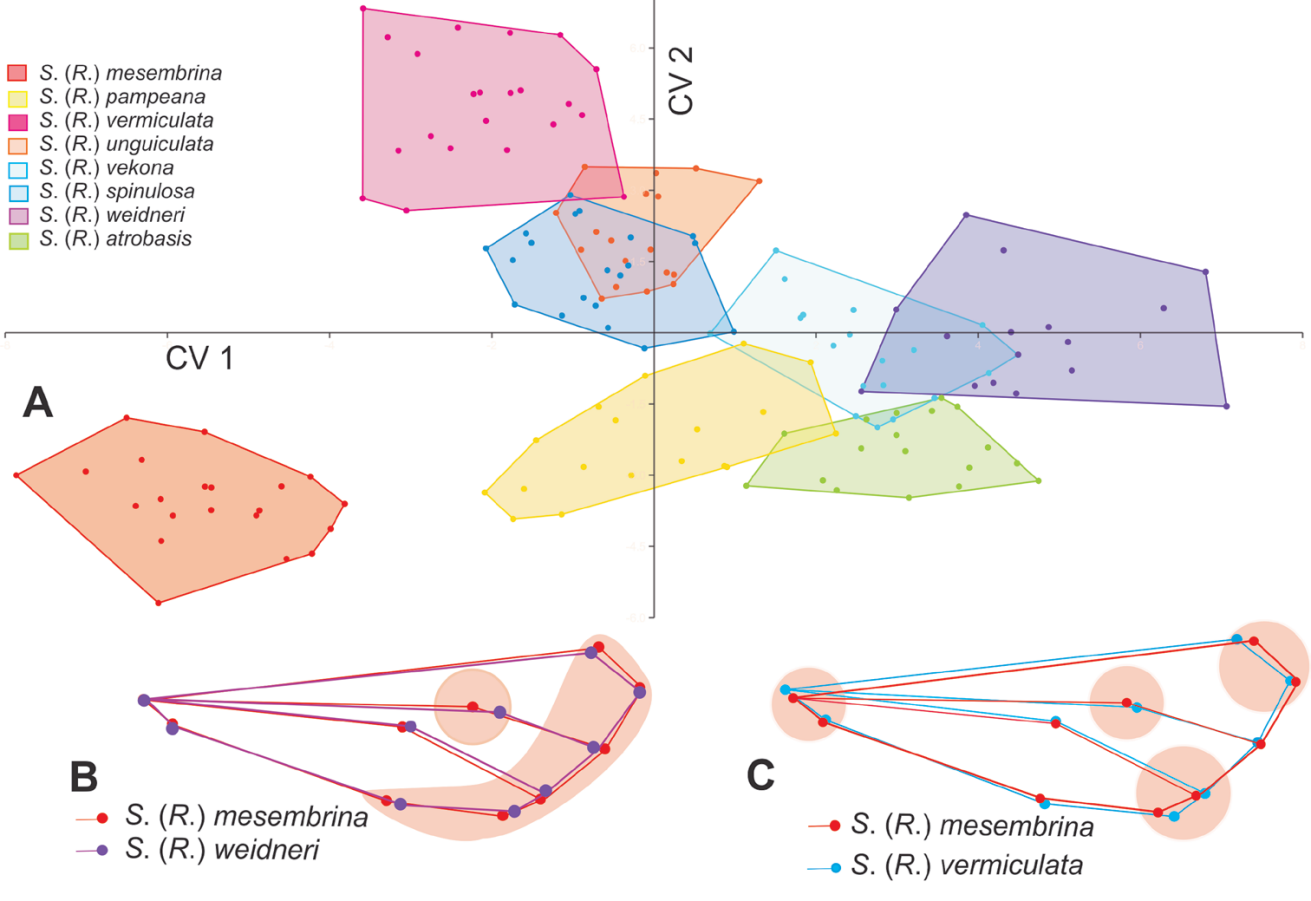
**A** Canonical Variate Analysis (CVA) scatter plot of the wing shape landmark data of eight *Smicridea* species **B, C** patterns of shape change along each axis by superposition of the mean configurations of the species located in extreme morphospaces of each axis **B** superposition of the mean (average) wings shape of Smicridea (Rhyacophylax) mesembrina - S. (R.) weidneri**C** superposition of the mean wings shape of S. (R.) mesembrina - S. (R.) vermiculata.

This approach allowed the differentiation of the analyzed species based on their wing shape. In the CVA analysis, the first two axes explained 79.25% of the shape variance. The shape configurations of *S.mesembrina* and *S.weidneri* occupied extreme morphospaces in the CV1 axis, and *S.mesembrina* and *S.vermiculata* in the CV2 axis (Fig. 8A). The superposition of the mean configurations of these species associated with CV1 and CV2 (Fig. 8B, C) showed that the basal bifurcation of M_1_ and M_2_ (landmark 8) and the apex of the wing (landmarks 3–5, 7, 9, 10) were the most affected areas for *S.mesembrina* and *S.weidneri* (Fig. 8B) while the base of the anal area (landmarks 1, 2), the apices of Cu_1a_ and M_4_ (landmarks 4, 5), the basal bifurcation of M_1_ and M_2_ (landmark 8), and the apex of R_3_ (landmark 10) were the most affected for *S.mesembrina* and *S.vermiculata* (Fig. 8C). CV1 was able to discriminate 4 groups: the first made up of *S.mesembrina*, the second by *S.pampeana* + *S.unguiculata* + *S.spinulosa*, the third by *S.vekona* + *S.atrobasis*, and finally *S.weidneri*. CV2 discriminated species that had not been separated by CV1. Although overlapping morphospaces were observed, the shape conformations were statistically different (p < 0.05) and a high percentage of correct reclassification of the specimens was obtained using the complete data set (83%), which increases if pairs of species are taken into consideration (Table 1).

**Table 1.** Canonical variate analysis of *Smicridea* species mean wing shape. Number of individuals used of each species are indicated in diagonal. The percentage of correct classification above the main diagonal and Mahalonobis distances are below. The P-values < 0.05 for permutation tests (2000 permutation runs) are marked with asterisks (*).

	*S.atrobasis*	*S.vekona*	*S.weidneri*	*S.spinulosa*	*S.unguiculata*	*S.vermiculata*	*S.mesembrina*	*S.pampeana*
*S.atrobasis*	18	100.00%	79.41%	100.00%	100.00%	100.00%	100.00%	100.00%
*S.vekona*	5.9096*	19	88.57%	86.48%	88.74%	97.56%	100.00%	91.89%
*S.weidneri*	4.0849*	4.9467*	16	100.00%	97.22%	100.00%	100.00%	97.05%
*S.spinulosa*	6.9183*	4.7454*	6.12*	18	92.10%	92.50%	100.00%	100.00%
*S.unguiculata*	6.1731*	5.1658*	6.2365*	2.8877*	20	92.85%	100.00%	92.10%
*S.vermiculata*	9.1635*	8.2066*	8.518*	4.5723*	4.3243*	22	100.00%	100.00%
*S.mesembrina*	9.1130*	9.3375*	10.8162*	8.0428*	7.1655*	9.1801*	21	97.44%
*S.pampeana*	5.1981*	5.1464*	6.3722*	5.3095*	4.6305*	7.9502*	6.4921*	18

**Figure 9.**
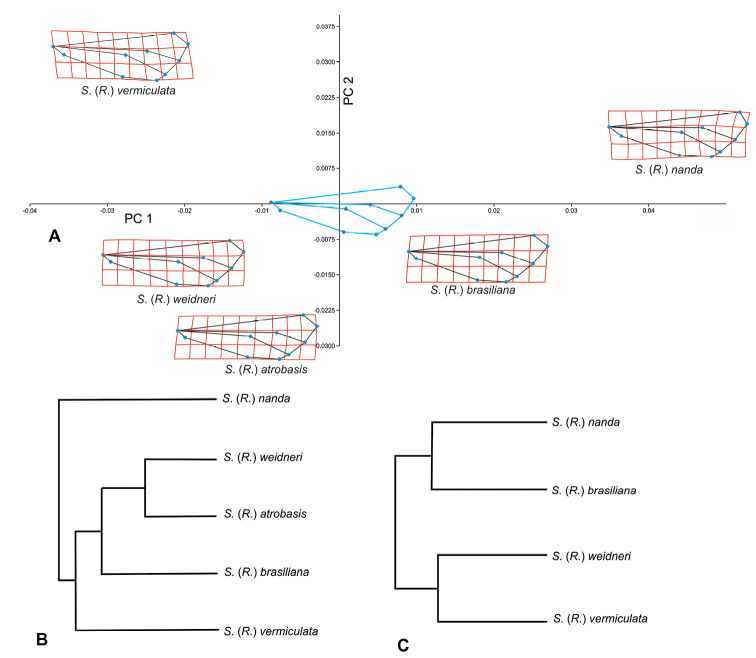
Principal Component Analysis (PCA) of the geometric landmark data of the wings of the *brasiliana* species group **A** scatter plot showing the average configuration of the wing shape in the deformation grids of each species from consensus shape of species group (indicate in the center of the plot) in the first two CPs. The circles represent the locations of the reference points in the mean shape of each species **B**UPGMA. Dendrogram from Mahalanobis distance of *brasiliana* species group and **C** without Smicridea (Rhyacophylax) atrobasis.

The principal component analysis of the consensus shapes of the species included in the *brasiliana* group revealed that *S.nanda* showed the furthest configuration and S. (R.) brasiliana the closest from the consensus shape of the group (Fig. 9A). The morphospaces that the different average configurations occupy in the space of the PCA plot can be visualized in Fig. 9A. *Smicrideaweidneri* and *S.atrobasis* showed the most similar configurations, although statistically different (p < 0.05). This result was contradictory with the morphological analysis of the genitalia and head that suggested that *S.atrobasis* was not closely related with the rest of the species in the group. Therefore, we performed two UPGMA a posteriori, the first including all the species of the *brasiliana* species group (Fig. 9B) and the second excluding *S.atrobasis*. In the latter UPGMA, both *S.weidneri* and *S.vermiculata* as well as *S.nanda* and *S.brasiliana* were grouped together (Fig. 9C).

### ﻿Wing size analysis

**Figure 10.**
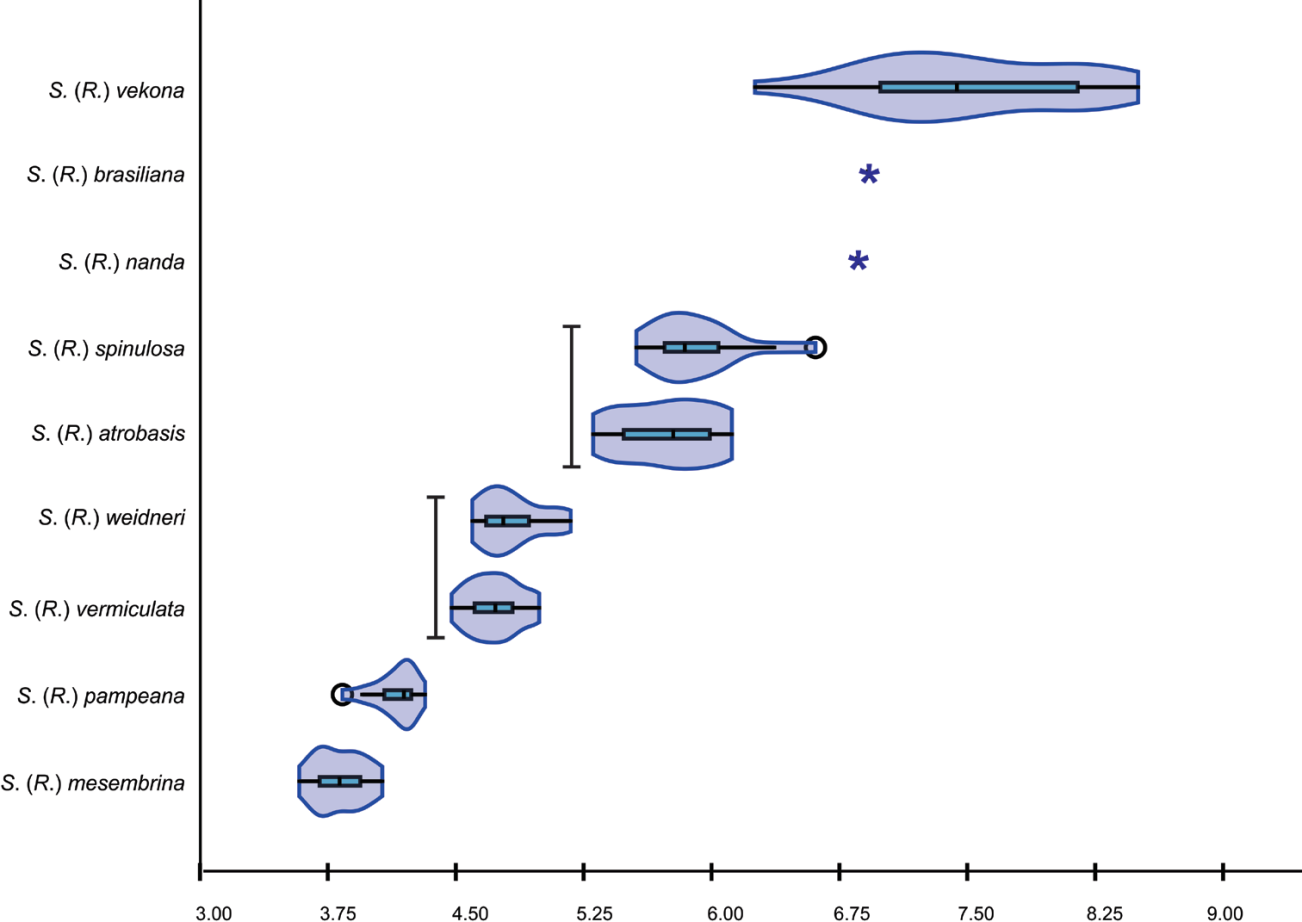
Violin plot of the centroid sizes (CS) of the wings of the ten *Smicridea* species analyzed. Bar: no significant differences between CSs (p > 0.05). *: *n* = 1.

All species included in the analysis differed in CS (p < 0.05), except S. (R.) spinulosa with S. (R.) atrobasis (p > 0.05) and S. (R.) vermiculata with S. (R.) weidneri (p > 0.05) (Fig. 10).

## ﻿Discussion

Based on the analysis of the fine structure of the genitalia of the species of the *brasiliana* group we consider that the group is heterogeneous. *Smicrideabrasiliana*, *S.weidneri*, and *S.nanda* seem to be closely related species with complex phallic structures, including internal plates of different shapes associated with the distal end of the sclerotized ejaculatory duct that are absent in *S.atrobasis* and *S.vermiculata*. In particular the genitalia of *S.vermiculata* is most similar to that of *S.dentifera* Flint and *S.unguiculata* Flint, which are unplaced to species groups, that have a simple ejaculatory duct and lateral subapical spines at the phallus. The analysis of the morphology of the heads of these species also supports a closer relationship between *S.brasiliana*, *S.weidneri*, and *S.nanda*, with anterolateral setose warts bifid and posterior setose warts triangular. The head of *S.vermiculata* is more similar in shape to these species but differs in the structure of the posterior setose warts that bear internal lobes and the postgenal areas that are more developed. In contrast, the head of *S.atrobasis* is unique in the group, with a more quadrangular outline, very large eyes, a reduced interocular area, and oval anterolateral setose warts. The genitalia of this species is also different from the rest: the apex of the ejaculatory duct is shaped like an arrowhead in dorsal view and the phallus ends in two laterodorsal and two lateroventral lobes. The presence of apicolateral lobes on the phallus is also found in S. (R.) mesembrina, not placed in a species group. The relationships between *S.brasiliana*, *S.weidneri*, and *S.nanda* are also strengthened by the configurations of the forewings, as was observed through the geometric morphometrics analysis.

The placement of *S.atrobasis* in the *brasiliana* group is conflictive. As stated before, although the configuration of the forewing of this species is similar to that of *S.weidneri*, the genitalia and the morphology of the head of both species differ. The relationships of the forewing configurations in the *brasiliana* species group were compared in the dendrogram, with and without *S.atrobasis*. The exclusion of this species from the analysis shows the same patterns that the ones observed using the morphology of the genitalia and features of the head. In this context, the similarities in the forewing configuration could be seen as a homoplasy rather than a homology, although further phylogenetic analyses are needed for confirmation.

The geometric morphometric analysis of wing shapes was useful for discriminating the species herein studied. This is the first study that uses this methodology in the order Trichoptera and needs to be examined in more species of *Smicridea*, and other caddisfly taxa as well, using not only wing shapes but other structures of the body. The larvae of Smicridea (Rhyacophylax) are good candidates to test this approach. In this subgenus the larvae are generally very similar, with no clear defining characters to separate them, but there are subtle interspecific differences in the shape of the head and the frontoclypeal apotome (JS pers. obs.). These differences in shape could be tested with this methodology. Taking into consideration that landmark configurations can be used in phylogenetic reconstructions (Catalano et al. 2010, 2015; Palci and Lee 2019), the exploration of this type of characters in the study of Trichoptera can be of great relevance.

## ﻿Conclusions

In this study, we provided a new approach for the delimitation of species in the genus. The head morphology is somewhat overlooked in the descriptions of most Trichoptera species. Here, we propose a more comprehensive approach including more detailed descriptions of relevant characters, besides the male genitalia, that would be useful for differentiating closely related species. Likewise, we demonstrated that the geometric morphometrics analysis of wing shapes can be used to discriminate the species of Smicridea (Rhyacophylax) herein studied. This fast, simple, and inexpensive method proved to be an efficient technique to confirm the identity of the specimens and could potentially be used to differentiate cryptic species, which were previously reported in *Smicridea* and other insect genera (Pauls et al. 2010; Chroni et al. 2018; Chatpiyaphat et al. 2021). Furthermore, it can also be a source of characters for phylogenetic analysis, not as a substitute for traditional morphological characters, but rather as a complementary descriptor of shape diversity (Palci and Lee 2019).

Even though the analyzed features indicate that the *brasiliana* group might not be a natural group as informally defined, the relationships between these species and the rest of the species in the subgenus Rhyacophylax must be established by a phylogenetic analysis and the monophyly of all the current groups of species should be tested.

## Supplementary Material

XML Treatment for Smicridea (Rhyacophylax) brasiliana

XML Treatment for Smicridea (Rhyacophylax) weidneri

XML Treatment for Smicridea (Rhyacophylax) vermiculata

XML Treatment for Smicridea (Rhyacophylax) atrobasis

XML Treatment for Smicridea (Rhyacophylax) nanda
